# Development of Biodegradable Composites Using Polycaprolactone and Bamboo Powder

**DOI:** 10.3390/polym14194169

**Published:** 2022-10-04

**Authors:** Satya Guha Nukala, Ing Kong, Vipulkumar Ishvarbhai Patel, Akesh Babu Kakarla, Wei Kong, Oliver Buddrick

**Affiliations:** 1 School of Computing, Engineering and Mathematical Sciences, La Trobe University, Bendigo, VIC 3550, Australia; 2 Centre for Foundation and General Studies, Infrastructure University Kuala Lumpur, Block 11, De Centrum City, Jalan Ikram-Uniten, Kajang 43000, Selangor, Malaysia; 3 Faculty of Higher Education, William Angliss Institute, Melbourne, VIC 3000, Australia

**Keywords:** bamboo powder, polycaprolactone, biodegradable, mechanical properties, thermal properties, hardness

## Abstract

The use of biodegradable polymers in daily life is increasing to reduce environmental hazards. In line with this, the present study aimed to develop a fully biodegradable polymer composite that was environmentally friendly and exhibited promising mechanical and thermal properties. Bamboo powder (BP)-reinforced polycaprolactone (PCL) composites were prepared using the solvent casting method. The influence of BP content on the morphology, wettability, and mechanical and thermal properties of the neat matrix was evaluated. In addition, the degradation properties of the composites were analysed through soil burial and acidic degradation tests. It was revealed that BP contents had an evident influence on the properties of the composites. The increase in the BP content has significantly improved the tensile strength of the PCL matrix. A similar trend is observed for thermal stability. Scanning electron micrographs demonstrated uniform dispersion of the BP in the PCL matrix. The degradation tests revealed that the biocomposites with 40 wt·% of BP degraded by more than 20% within 4 weeks in the acidic degradation test and more than 5% in the soil burial degradation test. It was noticed that there was a considerable difference in the degradation between the PCL matrix and the biocomposites of PCL and BP. These results suggest that biodegradable composites could be a promising alternative material to the existing synthetic polymer composites.

## 1. Introduction

The use of products made from polymers in the daily lives of individuals and society has increased due to their ease of production and relatively low cost and versatility [1,2]. However, this increase has led to the accumulation of an enormous amount of polymeric waste, which poses a serious hazard to the environment. Notably, most polymeric products are developed from non-biodegradable polymers, which can take years to degrade at the end of the product life cycle [3]. Therefore, researchers have been actively seeking ways to reduce the environmental impacts of polymeric products through the practice of reducing, reusing, and recycling [4,5]. Thus, there is a glowing interest in exploiting new eco-friendly materials based on biodegradable polymers to reduce environmental hazards [6].

Biodegradable polymers are materials that can degrade in the environment when exposed to various conditions such as temperature, ultraviolet radiation, humidity, and soil pH [7]. In addition, other ecological actions involving microorganisms (bacteria, fungi, and algae) aid the rapid degradation of polymers. As a result, biodegradable polymers can be transformed into water, carbon dioxide, or methane gas without causing environmental pollution [8]. Polycaprolactone (PCL) is a notable biodegradable polymer that can be obtained through the polymerisation of hydroxy caproic acid or by opening the ϵ-caprolactone ring [9]. PCL has good mechanical properties, such as flexibility and impact resistance [10], low glass transition and melting temperatures [11], and biocompatibility. Furthermore, PCL can degrade easily in a short period (a few months to many years), depending on its molecular weight, degree of crystallinity, shape, porosity, and the surrounding environment [12]. Therefore, PCL is used in numerous products, such as packaging materials, microcapsules for sustainable agriculture, and biomedical orthopedic applications [13]. The cost and complexity of the PCL manufacturing process greatly limit its large-scale applications.

One of the effective and economic methods to reduce the production cost of PCL is to partially replace the expensive polymer with low-cost fillers without sacrificing the biodegradation performance of PCL. Karakus et al. [14] studied the thermal and mechanical properties of PCL reinforced with wheat straw flour composites. The composites were produced by using the injection moulding technique. Adding wheat straw flour to the PCL matrix improved the flexural and tensile modulus but reduced the impact strength and elongation at break. Valdés et al. [15] evaluated the morphological, mechanical, thermal, and degradation properties of PCL-based biocomposites reinforced with almond skin. The results indicated that an increase of filler loading from 10 wt·% to 30 wt·% increases the elastic modulus from 280 MPa to 350 MPa. The improved thermal properties with increased filler content indicated effective adhesion between the matrix and filler. Furthermore, the presence of almond skin in PCL aided in accelerating the degradation rate of composites.

Bamboo is one of the most rapidly growing species of timber material. It possesses strong mechanical and thermal properties, and it has low nitrogen, sulphur, and ash content. According to Kitagawa et al. [16], 60% of the cellulose found in bamboo has a high concentration of lignin. Furthermore, bamboo has a small microfibrillar angle (2–10°). Because of these intrinsic properties, bamboo has been widely used in many applications such as fencing, furniture, roofing, clothing, and especially in construction and scaffolding in the construction industry. The high consumption of bamboo has led to the accumulation of waste bamboo in landfills. This sustainable waste material can be reused in the production of composites as reinforcement for various polymer matrices. Researchers have developed a range of methods to extract bamboo in the form of fibers, particulates, and powders [17,18,19].

Rasheed et al. [20] developed biodegradable composites with microcrystalline cellulose (MCC) extracted from bamboo chips reinforced with polylactic acid (PLA) and polybutylene succinate (PBS). The biocomposites were produced by melt-mixing at 180 °C and hot-pressing at 180 °C. The results stated that the addition of MCC enhanced the crystallinity, thermal stability, and tensile strength of the PLA-PBS blends. Xing et al. [21] studied the effect of the bamboo flour (BF) loading on the mechanical properties, thermal stability and, melt-crystallisation behaviour of a blend of PCL and PLA. BF-reinforced PCL-PLA composites were manufactured by blending BF and PCL–PLA using the melt-mixing process. The results revealed that the mechanical properties of composites first increased and then decreased with the increase in BF loading. The further impact strength, tensile, and elongation at the break of the composites achieved a maximum of 1.26 kJ/m^2^, 12.68 MPa, and 5.2%, respectively, when the BF mass fraction was 40%. There were no significant changes in the thermal properties with the increase in the BF mass fraction, although a change in the glass transition temperature and an increase in the crystallisation temperature were identified [21]. Chen et al. [22] examined the mechanical and rheological properties of PLA-polypropylene (PP) reinforced with bamboo fibers. The composites demonstrated a uniform dispersion of bamboo fibers in the matrix. Furthermore, the thermal and mechanical properties were strengthened when the filler loading was increased by up to 35%. Qi et al. [23] investigated a bamboo powder (BP)-reinforced PBS composite as a biofilm carrier for denitrification. The results indicated that the incorporation of BP in a PBS matrix achieved the acceptable removal of nitrates. An antibacterial analysis demonstrated that the interaction between the composite and bacterial and fungal communities played a vital role in composite degradation and denitrification.

Many studies focused on developing a novel material by mixing biodegradable polymers with natural fibers [24,25]. However, the open literature has provided very little attention to the combination of PCL and waste products of bamboo. Therefore, in this study, the bamboo powder was extracted from the waste products of bamboo after industrial processing and used as a filler to reinforce the PCL matrix. The composites were characterised using a scanning electron microscope (SEM) and Fourier transform infrared (FTIR) spectroscopy to observe the morphological and chemical interactions of the composites. In addition, the influence of bamboo powder on the thermal and mechanical properties of the PCL matrix is also analysed. Furthermore, the porosity, wettability, and biodegradability of the composites were assessed.

## 2. Materials and Methods

### 2.1. Materials

PCL, dichloromethane (DCM), and hydrochloric acid (HCl) were purchased from Sigma Aldrich (Melbourne, Australia), and the processed bamboo canes were supplied by Raw Boards Pty Ltd. (Bendigo, Australia). The canes were ground into a fine powder of 70–90 µm using a carpentry grinder.

### 2.2. Solvent Casting and Compression Moulding

**Figure 1:**
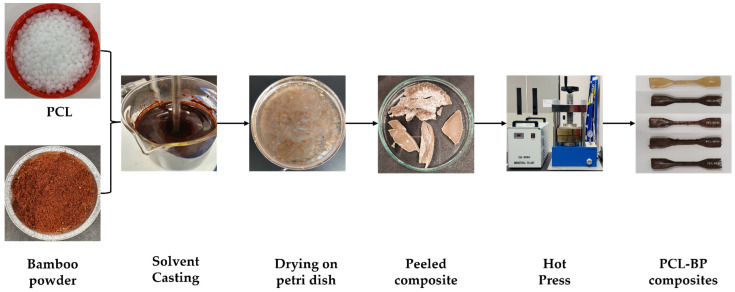
Illustration of the solvent casting of PCL and PCL-BP composites.

Initially, the BP was dried in the oven at 105 °C for 24 h to remove the moisture content. Subsequently, PCL and BP were weighed according to the weight percentage, as summarised in Table 1 [10]. The PCL was then dissolved in the DCM through mechanical stirring at a temperature of 50 °C and a speed of 500 rpm for 1 h, and the BP was slowly added to the solution, stirred for another 30 min to achieve a mixing consistency, and finally, the mixture was poured into a petri dish and oven dried at 40 °C for 24 h. The composite film was then peeled off from the petri dish and broken into small fragments and compressed using a hydraulic hot press at a temperature of 60 °C and a pressure of 20 kPa for 15 min. The illustration of the solvent casting of PCL-BP composites is shown in Figure 1.

### 2.3. Morphology

The morphology of the fractured cross-sectional surface of the PCL-BP composites was observed using an SEM (Hitachi Benchtop SEM 3030, Tokyo, Japan). The samples were vacuum sputter coated with platinum at 10 kV for 30 s before the microstructure was examined. The micrographs were obtained at 15 kV in a low vacuum mode.

### 2.4. Degradation

#### 2.4.1. Soil Burial Degradation

The PCL-BP composites were buried in wet fertile black soil for the soil biodegradation test [26]. The black soil consisted of hummus, which included crushed tree branches and leaves. The test was carried out at room temperature, and the relative humidity was 70–90%. The samples (5 cm in length) were buried in loose soil under the top-soil surface layer and weighed at regular intervals of 4, 8, 12, 16, 20, 24, and 28 days. The samples were washed with distilled water to remove any residual dirt and oven dried at 50 °C to measure weight loss [27,28]. Each composite was weighed before and after degradation, and the weight loss of each composite was calculated using Equation (1):

(1) $$M\left(\%\right)=\left[\left(M_{i}-M_{f}\right)/M_{f}\right]\times 100$$

where *M_i_* is the initial weight of the composite and *M_f_* is the final weight of the composite.

#### 2.4.2. Acidic Degradation

The PCL-BP composites were subjected to acidic degradation to evaluate their degradation in an acidic environment [26]. The degradation test was conducted in a glass bottle with a sample immersed in 50 mL of HCl. The sample was weighed at regular intervals of 2, 4, 6, 8, 10, 12, 14, 16, 18, 20, 22, 24, 26, and 28 days, and the average weight loss was calculated using Equation (2):

(2) $$W\left(\%\right)=\left[\left(W_{t}-W_{o}\right)/W_{o}\right]\times 100$$

where *W_t_* and *W_o_* are the specimen weights before and after immersion in acid, respectively. Each sample was performed thrice, and the mean and standard deviation (SD) were reported.

### 2.5. Porosity

The porosity percentage of the PCL-BP composites was evaluated based on Archimedes’ principle using a specific gravity bottle [29,30]. The porosity of the composites was calculated based on Equation (3):

(3) $$Porosity\left(P\%\right)=\frac{w_{2}-w_{3}-w_{s}/\rho_{e}}{w_{1}-w_{s}/\rho_{e}}\times 100$$

where *w_1_* is the specific gravity bottle weight filled with ethanol, *w_2_* is the specific gravity bottle weight with ethanol and sample, *w_3_* is the specific gravity bottle weight after the ethanol-saturated sample has been removed, *w_s_* is the sample weight, and *ρ_e_* is the ethonal density. Each sample was performed thrice, and the mean and standard deviation (SD) were reported.

### 2.6. Water Contact Angle Measurement

The contact angle of the PCL-BP composites was measured using the sessile-drop technique. A micrometre syringe was used to displace a droplet on the surface of the PCL-BP composites, and the contact angle was measured by scanning the droplet profile for 15 s with an Attention Theta Flex instrument (Biolin Scientific, Gothenburg, Sweden), following the procedure used in other studies [31,32]. The water droplet size was maintained at approximately 3 μL to avoid the effects of weight [33]. The measurements were repeated three times (*n* = 3) for each sample.

### 2.7. Thermal Analysis

#### 2.7.1. Thermogravimetric Analysis

A thermogravimetric analysis (TGA) was performed using a PerkinElmer TGA 4000 (Waltham, MA, USA). Samples of approximately 4 mg were heated from 30 °C to 850 °C at a rate of 10 °C/min under a nitrogen flow of 20 mL/min.

#### 2.7.2. Differential Scanning Calorimetry

Differential scanning calorimetry (DSC) measurements were performed using a PerkinElmer DSC 6000 (Waltham, MA, USA). First, the samples were heated from 30 °C to 200 °C at a rate of 20 °C/min and then retained at that temperature for 5 min to remove the thermal history. The samples were then cooled to 30 °C at a rate of 20 °C/min and heated again to 200 °C at a rate of 20 °C/min. The endothermic peaks were recorded as the enthalpy of melting (ΔH_m_) and melting temperature (T_m_).

### 2.8. Fourier Transform Infrared Spectroscopy

The FTIR spectra of the PCL-BP composites were recorded from 4000 to 650 cm^−1^ with the help of a computerised FTIR (Cary 630, Agilent Technologies, Santa Clara, CA, USA). The samples had been air-dried before the analysis. The transmission method used 32 scans with a 4 cm^−1^ resolution [34].

### 2.9. Mechanical Properties

#### 2.9.1. Tensile Properties

The tensile properties of the composites were measured using a Zhongli ZL-8001A universal testing machine (Dongguan Zhongli Instrument Technology Co., Ltd., Dongguan, China) at a crosshead speed of 3 mm/min and a load of 500 kN, with the samples compressed using a hot press into dog-bone shape (ASTM D638 Type 4). The samples were in dry condition before testing and were tested at room temperature. Each sample was performed thrice, and the mean and standard deviation (SD) were reported.

#### 2.9.2. Flexural Properties

The three-point bending behaviour of the PCL-BP composites was determined through three-point flexural testing using an Instron 5890 (Norwood, MA, USA) universal testing machine. The test was performed at 5% deflection of the composite samples [35,36]. The samples were prepared using hot press according to ASTM standards (ASTM D790) [37].

### 2.10. Hardness Test

The hardness test was conducted using Vickers hardness (DuraScan 32 series G5, Emco-Test, Kuchl, Austria). A 0.2 HV Vickers indenter was used to perform the measurement. Initially, a minor load of 5 kg was applied to ensure that the indenter penetrated the specimen, eliminating possible surface roughness errors. A major load of 10 kg was then applied for 10 s, and the hardness value was denoted in HV. The measurements were repeated three times (*n* = 3) for each sample.

### 2.11. Statistical Analysis

Statistical analysis was evaluated using GraphPad Prism 9.0 (GraphPad Software, Inc., San Diego, CA, USA) using the ANOVA method. The calculations were carried out three times (*n* = 3) for each sample and presented as mean ± standard deviation (SD) unless otherwise stated. The standard error of the mean is represented by error bars in all the figures, and the significance level of the *p*-value of ≤0.05 was determined to be significant (*). The analysis was performed based on the following literature [29,38,39].

## 3. Results and Discussion

### 3.1. Morphology

**Figure 2:**
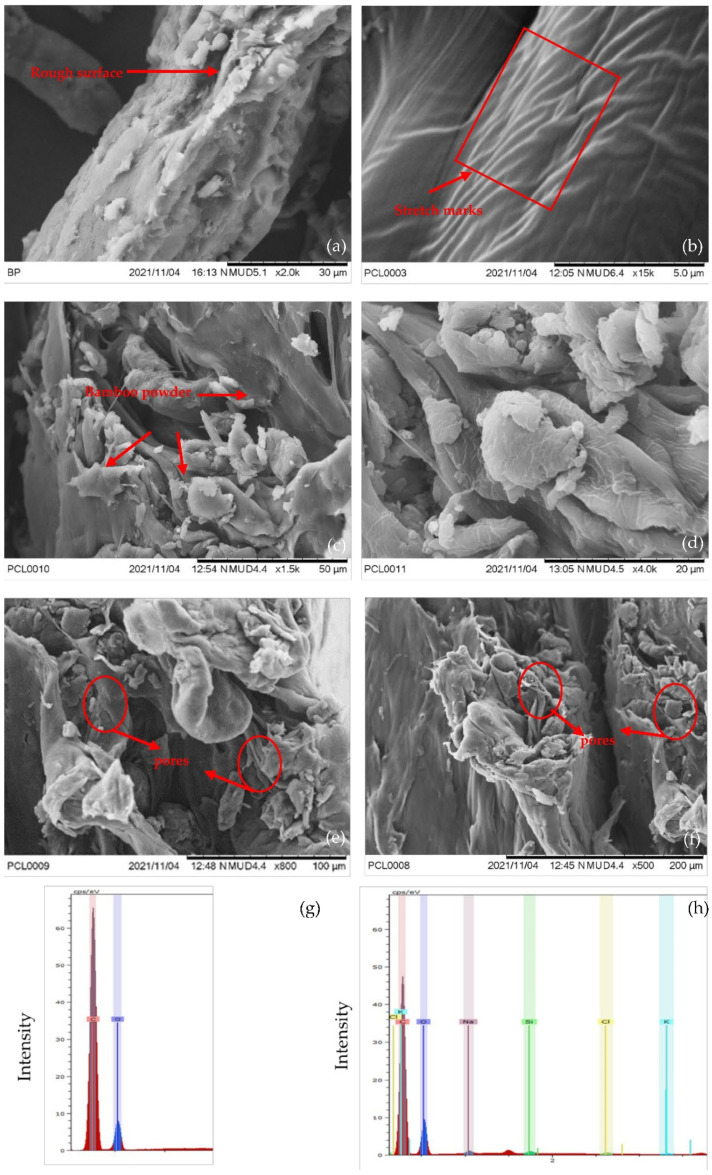
SEM micrographs of (**a**) BP; (**b**) PCL; (**c**) PCL-BP30; (**d**) PCL-BP40; (**e**) pores of PCL-BP30; (**f**) pores of PCL-BP40; EDX spectra of (**g**) PCL and (**h**) PCL-B40.

The SEM micrographs of BP and PCL-BP composites are presented in Figure 2. BP exhibited a rough surface (Figure 2a), which would help it to enhance the bonding between fiber and matrix [6]. In Figure 2b, the hot-pressed PCL showed a smooth and homogenous appearance. In addition, the stretch marks corresponded to the rupture of the specimen during the tensile test. Similar morphology was observed by Da Silva et al. [40]. Figure 2c,d shows the PCL-BP30 and PCL-BP40 composites, and it is evident that the BP was randomly dispersed in the PCL matrix [41]. In spite of the random dispersion of the BP, the PCL-BP composite exhibited some pores, as depicted in Figure 2e,f, which was evaluated using the porosity of the composite. Figure 2g,h depicts the energy dispersive X-ray (EDX) spectra of PCL and PCL-BP40. The carbon (C) and oxygen (O) peaks were expected to originate from the elemental composition of PCL. In the case of the PCL-BP40 composite, peaks of C and O were present, along with slight traces of sodium, potassium, chlorine, and silicon. The presence of the other elements may be caused by the contamination of the solvent during the solvent casting and hot-press procedures [34].

### 3.2. Fourier Transform Infrared Spectroscopy

**Figure 3:**
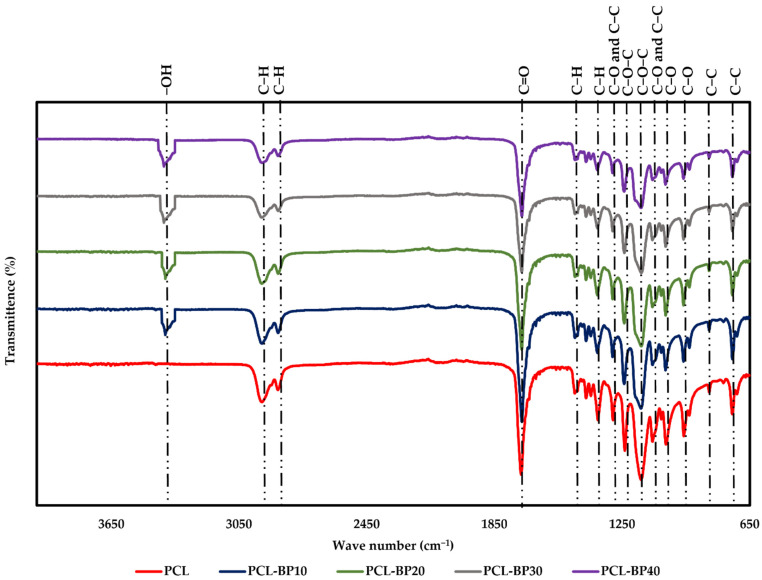
FTIR spectra of PCL and PCL-BP composites.

The FTIR spectra of the PCL and PCL-BP composites are presented in Figure 3. In the spectra of PCL, the weak peaks observed at 2940 cm^−1^ and 2870 cm^−1^ corresponded to asymmetric elongation of the methylene-oxygen (CH_2_-O) and symmetric methylene groups (CH_2_), respectively [42,43]. The sharp and strong peak representing C=O bonds was observed at 1720 cm^−1^ [44]. Furthermore, the peaks at 1292 cm^−1^ were related to the stretching of the C-C and C-O bonds in the crystalline phase [45]. In addition, the peaks at 1165 cm^−1^ and 959 cm^−1^ correspond to the C-O-C bond and stretching of the oxime bond in PCL [43]. In the spectrum of the PCL-BP composite, an absorption band was observed at 3350–3250 cm^−1^, which was assigned to typical OH stretching vibrations due to the contributions from -OH groups of the BP [46,47]. It can also be seen that when the BP content decreased, the band weakened [45,46]. Moreover, the peaks in the spectra of PCL-BP were similar to those of PCL, implying that there were no major structural changes due to the addition of BP in PCL, such as new chemical bond formation between the PCL chains and BP [43,48]. These spectra are consistent with those reported by Si et al. [11] and Vidal et al. [49].

### 3.3. Porosity

**Figure 4:**
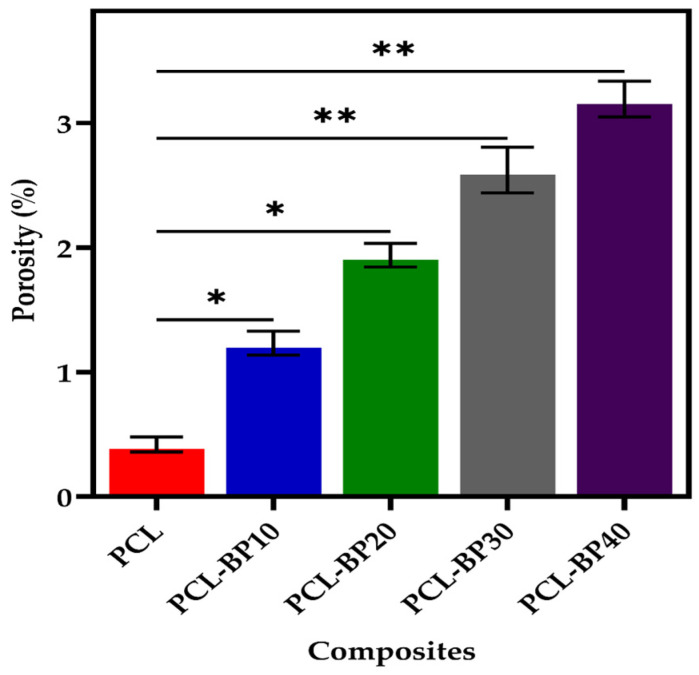
The porosity of PCL and PCL-BP composites (*n* = 3, * *p* ≤ 0.05, ** *p* ≤ 0.01).

The porosity of the PCL-BP composites is depicted in Figure 4. It can be seen from the figure that the increase in weight percentage of the BP increased the porosity of the PCL-BP composites. PCL had the lowest porosity at 0.41%, while the porosity of the PCL-BP composites increased from 1.3% to 3.25% with the increase of BP content from 10 wt·% to 40 wt·%. The increase in porosity is due to the reduction in the overall density of the composites with the increase in BP content. Swain et al. [50] reported that the incorporation of natural fibers into a biodegradable polymer led to an increase in water absorption, which directly affected the porosity of the composites. This is because the natural fibers mainly consist of cellulose, hemicellulose, lignin, and other natural proteins, which have high water-retaining capacities [51], and hence, more water-absorbing pores can be formed.

### 3.4. Water Contact Angle

**Figure 5:**
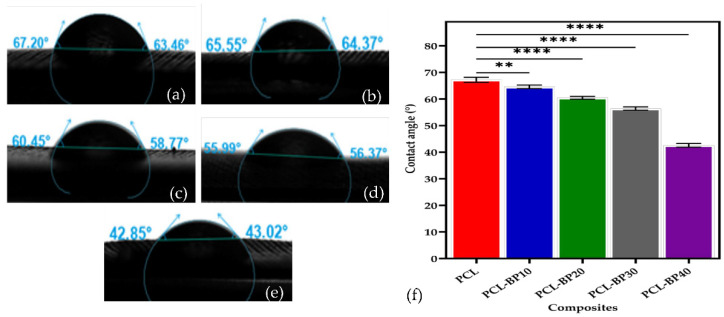
Sessile drop images of the PCL and its composites; (**a**) PCL; (**b**) PCL-BP10; (**c**) PCL-BP20; (**d**) PCL-BP30; (**e**) PCL-BP40; and (**f**) the contact angle measurements (*n* = 3, ** *p* ≤ 0.01, **** *p* ≤ 0.0001).

Figure 5a–e presents the sessile drop images on the composite surfaces to evaluate the water contact angle, and Figure 5f illustrates the contact angle measurements of PCL-BP composites. According to the literature, a contact angle below 90° indicates a good wetting surface for any liquids [52,53]. Figure 5 summarises the results, with the PCL surface having a water contact angle averaging approximately 65.33°, indicating partial wetting [54]. The water contact angle measurements for PCL-BP10 were 64.96°, followed by 59.58° for PCL-BP20, 56.18° for PCL-BP30, and 42.93°, the lowest water contact angle, for PCL-BP40. With the addition of BP, the water contact angle decreased due to the hydrophilic nature of BP. Wu et al. [55] studied the wettability of bamboo fiber-reinforced PLA composites. It was found that the contact angle decreased with the increase in bamboo fiber content in the composite. This could be due to the hydrophilicity of the bamboo fiber, which plays a vital role in decreasing the contact angles. The present study depicts a similar result as mentioned in the literature [55].

### 3.5. Degradation

#### 3.5.1. Acidic Degradation

**Figure 6:**
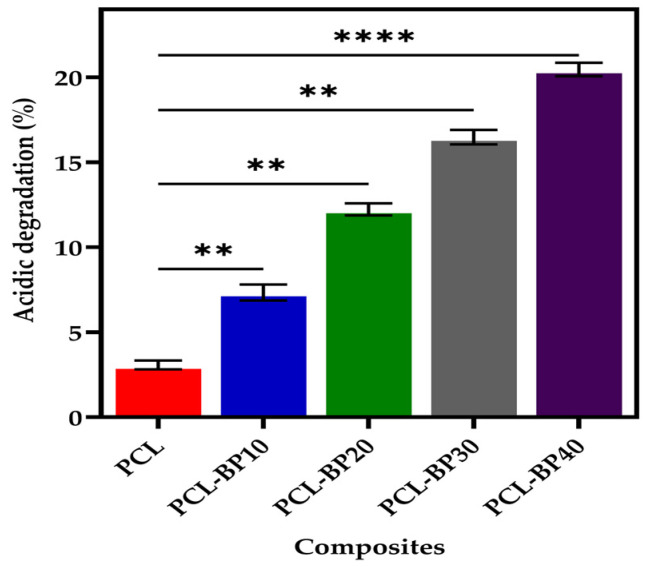
Acidic degradation of PCL and PCL-BP composites after 28 days of immersion in acidic medium (*n* = 3, ** *p* ≤ 0.01, **** *p* ≤ 0.0001).

The acidic degradation and composites of PCL are presented in Figure 6. The composites were subjected to acidic degradation for 28 days. The degree of PCL weight loss increased with the number of days. In terms of the composites, PCL-BP40 degraded the most, followed by PCL-BP30, PCL-BP20, PCL-BP10, and PCL. It can be observed that the degree of weight loss increased with the increase in BP content over the same period. PCL showed the least weight loss, ranging up to 2.9% throughout the acidic degradation test period, whereas PCL-BP40 showed the highest degradation, with 20.5% of weight loss after 28 days of immersion in an acidic medium. It was followed by PCL-BP30, PCL-BP20, and PCL-BP10 showing weight losses of around 16.5%, 12.2%, and 7.3%, respectively. It was reported by Yang et al. [26] and Abdul Khalil et al. [6] that the BP contains certain chemical contents such as some amount of starch and many hydroxyl groups, which are very hydrophilic in nature. This hydrophilic nature of BP possesses a strong degradation ability when immersed in an acidic medium. The degradation studies were carried out according to Khalil et al. [6]. As a result of the increase in BP, the degradation of the composites increased, as noted in the studies by Dinesh et al. [56], Lyu et al. [43], and Schrip et al. [57].

#### 3.5.2. Soil Burial Degradation

**Figure 7:**
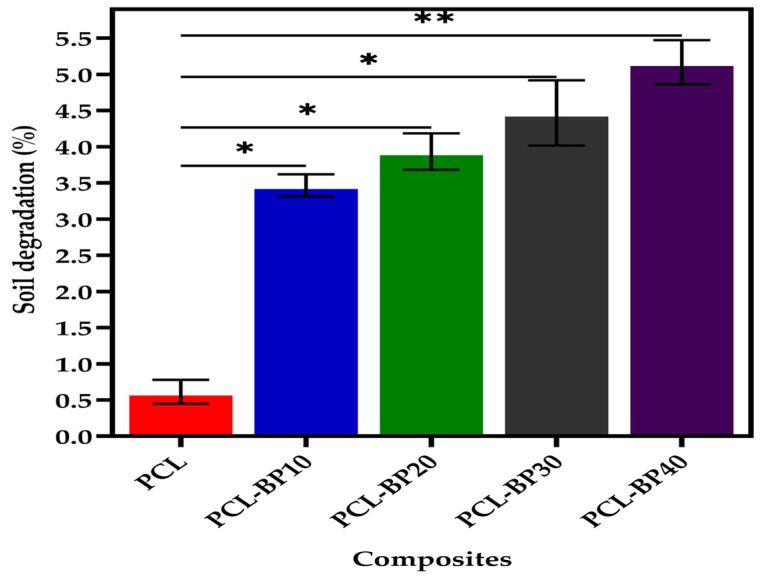
Soil burial degradation of PCL and PCL-BP composites (*n* = 3, * *p* ≤ 0.05, ** *p* ≤ 0.01).

The biodegradation of the composites during the soil burial period occurred because of the presence of moisture and other enzymatic actions involving microorganisms, leading to the weight loss of the composite material [28]. Figure 7 represents the weight change of the PCL and its composites after they were subjected to soil burial degradation for 28 days. The weight loss of the composites was more significant than expected with the increase in soil burial time. Higher loadings of BP led to greater weight loss after 28 days. Among all the composites, PCL-BP40 demonstrated the highest biodegradability, with 5.1% of weight loss after 28 days of soil burial. PCL-BP30, PCL-BP20, and PCL-BP10 demonstrated weight losses of 4.5%, 3.9%, and 3.5%, respectively, after 28 days of soil burial. However, the weight loss of the pure PCL was minimal, ranging up to 0.6% throughout the soil burial test period. The chemical contents, such as cellulose, hemicellulose, and lignin, and the hygroscopic features promoted the microbial activity in the composites during the soil burial test, leading to their weight loss [28].

In another study, Chee et al. [27] investigated the biodegradability properties of bamboo and kenaf fiber-reinforced epoxy composites. It was reported that the weight loss increased with the increase in fiber loading, exposure time, and soil burial time. In the present study, the outcome of the soil burial degradation test aligns well with that reported by Chee et al. [27], as with the increase in soil burial time and fiber loading, the weight loss percentage increased.

### 3.6. Mechanical Properties

#### 3.6.1. Tensile Properties

**Figure 8:**
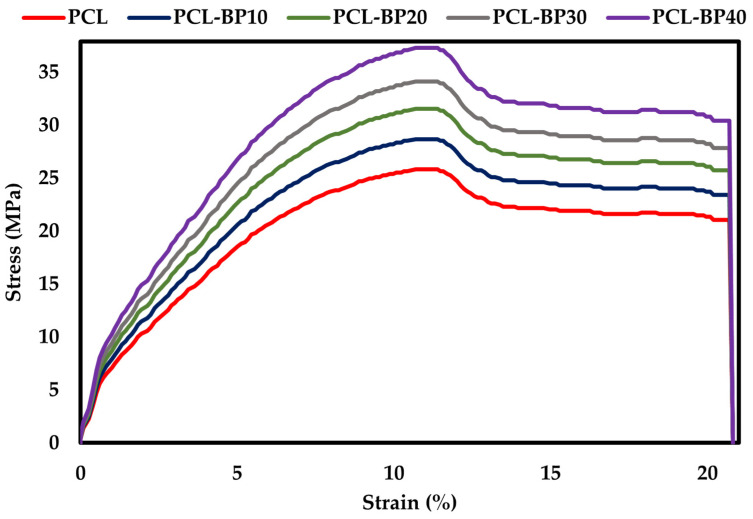
Tensile curve of PCL and PCL-BP composites.

Figure 8 presents the tensile stress-strain curves of the PCL and PCL-BP composites. The tensile strength measurements were conducted following those reported by Idicula et al. [58] and Mohanty et al. [59]. The measurements were carried out at 3 mm/min crosshead speed in order to obtain the rupture within the timeframe [60]. Tensile strength was found to increase with the increasing concentration of BP, as reported by Allaf et al. [54]. PCL had a tensile strength of up to 25.82 MPa, whereas PCL-BP10, PCL-BP20, PCL-BP30, and PCL-BP40 exhibited stress concentrations of 28.67, 31.54, 34.12, and 37.27 MPa, respectively. The mechanical properties of the composites are summarized in Table 2, demonstrating that Young’s modulus increased with the increase in BP content. According to Bhagabati et al. [10], PCL reinforced with 30% bamboo root flour revealed the highest tensile strength. Similarly, Yang et al. [26] reported that the tensile strength of bamboo fiber-reinforced PP composites increased with the content of fiber. Thus, the addition of BP was sufficient to reinforce the PCL for easy stress transfer and improved tensile strength, as reported in the following literature [61].

#### 3.6.2. Flexural Properties

**Figure 9:**
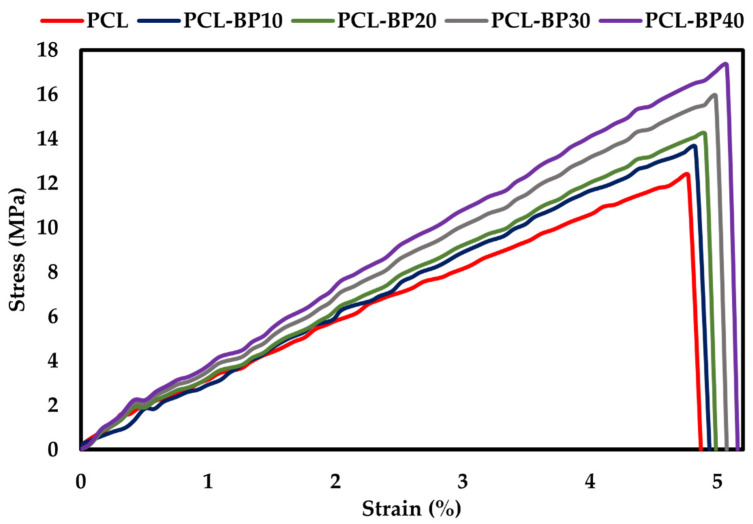
Flexural curves of PCL and PCL-BP composites.

The flexural behaviour was measured for the 5% deflection as per the ASTM D790 standard. The PCL-BP composite results were presented in Figure 9. The flexural strength increased with the increase in BP because of the even dispersion of BP in the PCL matrix, as observed in SEM micrographs. The PCL-BP40 had a flexural strength of 17 MPa, while the flexural strengths for the PCL-BP30, PCL-B20, and PCL-B10 were 16 MPa, 14 MPa, and 13 MPa, respectively. The pure PCL had the lowest flexural strength at 12 MPa. Campaña et al. [62] studied the mechanical properties of PLA reinforced with bamboo. It was reported that the 75% PLA and 25% BP composite demonstrated the highest flexural stress and flexural modulus [62]. It was also noted that the flexural strength increased with the increase in filler concentration. The results of the present study are consistent with those of the aforementioned study.

### 3.7. Hardness

**Figure 10:**
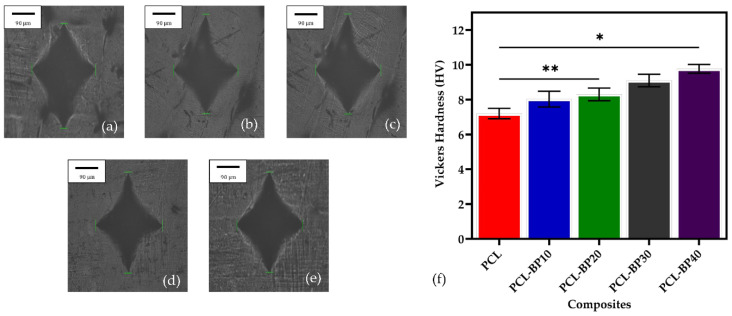
Images of hardness for PCL and PCL-BP composites: (**a**) PCL; (**b**) PCL-BP10; (**c**) PCL-BP20; (**d**) PCL-BP30; (**e**) PCL-BP40; and (**f**) Vickers hardness values (*n* = 3, * *p* ≤ 0.05, ** *p* ≤ 0.01).

The Vickers microhardness indentation optical images of the PCL-BP composites are shown in Figure 10a–e, and the Vickers hardness values are presented in Figure 10f. The Vickers hardness values significantly increased with the increase in BP. This is consistent with the studies reported by Kaymakci et al. [63] and Kord et al. [64]. A steady increase in the hardness values from PCL-BP10 to PCL-BP40 was observed. The continued rise in hardness values is due to the even dispersion of BP in the PCL matrix and improved interfacial adhesion between the PCL and BP, leading to fewer micro-voids and less filler debonding in the interphase region, which was reported in the following literature by Kaymakci et al. [65]. For instance, PCL had the lowest hardness value (7.2 HV) followed by PCL-BP10, PCL-BP20, and PCL-BP30 with 8 HV, 8.4 HV, and 9 HV, respectively, and PCL-BP40 had the highest hardness value (9.8 HV). These results are consistent with the study by Jumadi et al. [66].

### 3.8. Thermal Properties

#### 3.8.1. Thermogravimetric Analysis

**Figure 11:**
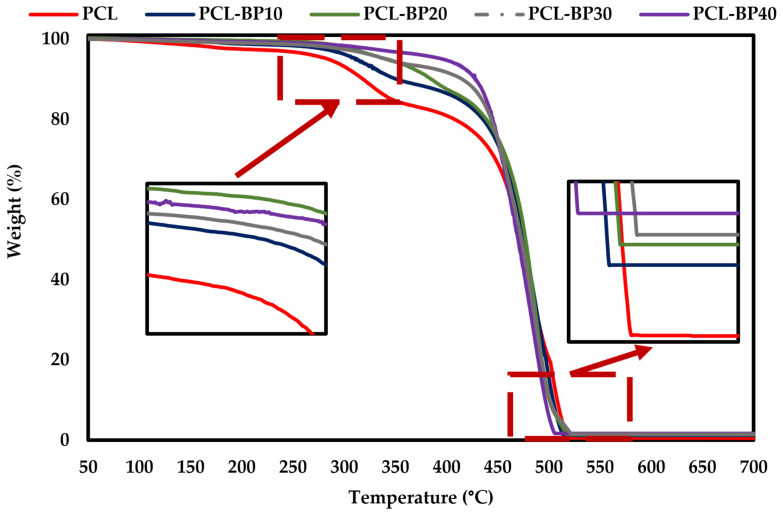
TGA thermograms of PCL and PCL-BP composites.

The TGA curves of the PCL and PCL-BP composites are presented in Figure 11. The thermal degradation of pure PCL is displayed in a two-step decomposition process. The first step of decomposition occurred at a temperature below 200 °C and was related to the evaporation of moisture in the composites. The second step occurred at a temperature above 350 °C. This second weight loss was associated with the degradation of the main polymer chains of neat PCL. Unlike PCL, the decomposition of PCL-BP occurred in three steps [67]. It was obvious that the first step below 200 °C was associated with the humidity and moisture evaporation from the composites [68]. The second step occurred between 200 and 475 °C and was related to the decomposition of the main components of BP such as cellulose, hemicellulose, and lignin [69,70,71]. The third step from 480 °C onward to 700 °C was associated with the degradation of residual lignin in PCL-BP composites [72]. The decomposition trends were similar to El Mechtali et al. [73], Rojas-Lema et al. [74], and Açıkalın et al. [75]. At higher temperatures (>500 °C), the presence of carbonated residuals in PCL-BP that were formed due to depolymerization, decomposition, and decarboxylation of hemicellulose and cellulose was noted. Compared to neat PCL, the thermal stability of PCL-BP increased with the addition of BP to the matrix. The thermal stability sequence is as follows: PCL-BP40 > PCLBP30 > PCL-BP20 > PCL-BP10 > PCL. Similar observations were reported for the other wood polymer composites (WPCs) [76,77].

#### 3.8.2. Differential Scanning Calorimetry

**Figure 12:**
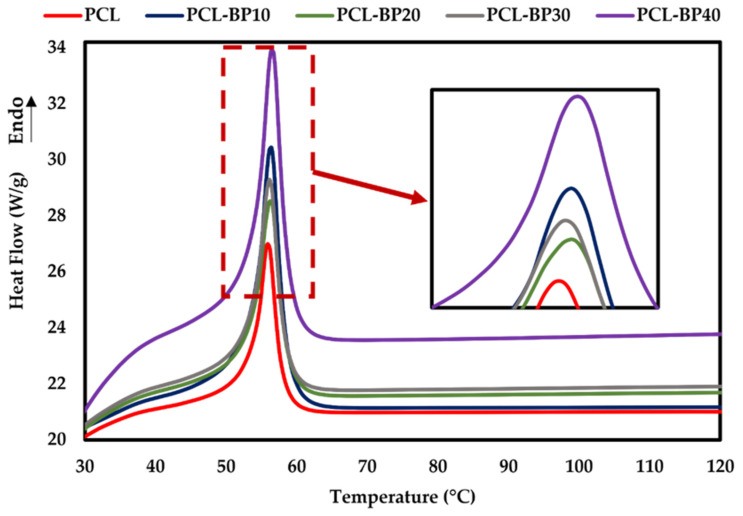
DSC curves of PCL and PCL-BP composites.

The DSC scans of the PCL-BP composites are presented in Figure 12. The enthalpy of melting (ΔH_m_) and melting temperature (T_m_) of the PCL-BP composites were identified and tabulated in Table 3. It can be seen that T_m_ increased slightly with the BP content in the PCL matrix. This was mainly caused by the expansion of the BP, which would have loosened the PCL structure as a result of the increased T_m_. PCL-BP40 had the highest melting temperature at approximately 57 °C, followed by PCL-BP30, PCL-BP20, and PCL-BP10 at 56 °C, and finally PCL at 55 °C.

The ΔH_m_ value is used as an indicator of the crystallinity of the composite. The ΔH_m_ for the PCL-BP composites decreased with the increase in BP content, resulting in a lower degree of crystallinity, as shown in Table 3. These results are similar to those obtained for BF-reinforced polypropylene composites in the study by Mi et al. [78]. The primary reason for the decrease in the crystallinity of the PCL-BP composites was the hindered motion of the PCL polymer in segments, mainly resulting from the presence of the BP in the composites. This is a result of the steric effect, with PCL being hydrophobic and BP being hydrophilic, leading to good dispersion in the fabrication of composites [46,79]. Similar observations were reported for the other types of WPCs [73,80,81].

## 4. Conclusions

PCL-BP-reinforced composites were fabricated using solvent casting and compression moulding techniques. The SEM micrographs revealed the even dispersion of PB in the PCL matrix and good interfacial adhesion between the PCL and BP. FTIR results demonstrated that all the individual peaks were related to the PCL-BP composites, indicating no structural changes or chemical bond formations. The biodegradable composites with comparable mechanical properties were obtained, where the tensile strength of the composites increased from 25.82 to 37.27 MPa, and the flexural strength increased from 15 to 23 MPa, with the increase in BP content, highlighting the strength and ductility of the composite with the addition of BP. Furthermore, the hardness of the composites increased from 7 to 9.8 HV with an increase in BP content from 0 wt·% to 40 wt·%. In terms of the thermal properties, it can be noticed that thermal stability increases along with a decrease in decomposition residue, and the melting enthalpy shows a drastic drop from 70 to 25 J/g with the increase in BP content in the PCL-BP composites. The BP content demonstrated the influence of degradation in natural and controlled environments. The degradation of composites increased with the increase in BP, and the degradation rate depended on the number of days. Since BP is hydrophilic in nature, the porosity and wettability increased with the increase in BP concentration (30 and 40 wt·%). The present work demonstrated the development of biodegradable composites with comparable mechanical and thermal properties to synthetic polymer composites, which could be an alternative material to the existing synthetic polymer composites.

## Figures and Tables

**Table 1:** Composition of PCL-BP composites.

Composite	PCL (wt·%)	BP (wt·%)
PCL	100	0
PCL-BP10	90	10
PCL-BP20	80	20
PCL-BP30	70	30
PCL-BP40	60	40

**Table 2:** Mechanical properties of PCL and PCL-BP composites.

Composite	Young’s Modulus (MPa)	Ultimate Tensile Strength (MPa)
PCL	3.01	25.82
PCL-BP10	3.17	28.67
PCL-BP20	3.51	31.54
PCL-BP30	3.92	34.12
PCL-BP40	4.19	37.27

**Table 3:** Melting temperature (T_m_) and enthalpy of melting (ΔH_m_) of PCL-BP composites.

Sample	Melting Temperature T_m_ (°C)	Enthalpy of Melting ΔH_m_ (J/g)
PCL	55	70
PCL-BP10	56	45
PCL-BP20	56	40
PCL-BP30	56	35
PCL-BP40	57	27

## Data Availability

Not applicable.
